# Nutritional status and health of community-dwelling older adults in urban areas of the Lalitpur district, Nepal

**DOI:** 10.1371/journal.pone.0344599

**Published:** 2026-03-18

**Authors:** Eebaraj Simkhada, Aerusha Simkhada

**Affiliations:** 1 Department of Internal Medicine, Manmohan Memorial Medical College and Teaching Hospital, Kathmandu Metropolitan City, Nepal; 2 Aditya College of Pharmacy, Jawaharlal Nehru Technological University, Kakinada, Andhra Pradesh, India; University of California San Diego, UNITED STATES OF AMERICA

## Abstract

**Background:**

Poor nutrition causes ill health and functional dependence in the older population. Different aspects of older people’s health and nutritional status are little known in the growing older population in Nepal. This study describes the nutritional status, socio-demographic factors, and health-related characteristics among community-dwelling older people in urban areas of Lalitpur district of Nepal.

**Methods:**

The study was a cross-sectional population-based study among older people aged 60 years and older, in five Village Development Committees (VDCs) around the outskirts of Lalitpur Metropolitan City of Lalitpur district of Nepal. Multi-stage cluster sampling with probability proportional to size was used to select a sample of 360 old aged individuals. Nutritional status was assessed by the Mini Nutritional Assessment (MNA) tool. The data on socio-demographic factors, socioeconomic factors, and health characteristics were presented in percentages, means, and standard deviations. Chi-square and ANOVA tests were performed to assess the associations of variables with malnutrition.

**Results:**

Using the MNA score, this study found 13% malnutrition and 45% at risk of malnutrition among participants. About 28% of the study population was overweight with a body mass index (BMI) > 25.0. About one-fourth (25.6%) were totally dependent financially on their children. Approximately 62.2% were diagnosed with chronic diseases. Hypertension was the most prevalent disease (26.5%), followed by Diabetes Mellitus. About 28.6% reported insomnia, and 18.6% of individuals reported having chronic pain. Agriculture as an occupation, increased age, hospitalization within a year and having insomnia were significantly associated with nutritional status in bivariate analysis.

**Conclusions:**

About half of the population had poor nutritional status. Old-aged individuals were suffering from chronic diseases, chronic pain, insomnia, and financial dependency. This study indicates an urgent need to take care of health of the older people with timely intervention at the community and national level.

## Introduction

Nutrition is crucial for the health and development of an individual [1]. Poor nutritional status is a common problem in older people. The well-being of the older adult can be adversely affected by malnutrition, causing a steady decline in functional status and susceptibility to complications associated with chronic illnesses. Malnutrition potentially impacts morbidity, mortality, and quality of life in older individuals [2].

### Global burden of malnutrition

An estimate indicated that about 805 million people were chronically undernourished globally in 2012–14 [3]. Undernutrition is highly prevalent in developing regions, and older adults are more likely to be affected by malnutrition than young persons.

The population of the world is becoming older as a consequence of a decline in fertility and increased life expectancy. It is projected that by the year 2050, the population of persons over 60 years will be two billion [4]. Aging should be taken as a privilege and a societal achievement. However, national health policies and programs give less priority to the health of the old-aged population.

Developing countries are aging faster than developed countries. As in most other countries, the proportion of older people is increasing in Nepal with an annual old-aged population growth rate of 3.39% [5]. Indeed, population aging can have major implications on health and social services, as increased age results in a greater likelihood of having a disability and needing assistance [6], since malnutrition is a risk factor for the health and wellbeing of old people. In a study, old-aged persons, health problems, and increased age had a negative impact on nutritional status. Educational status and less expenditure on food were associated with poor nutritional status among the older individuals [7].

Poor nutrition causes ill health in the older population. Increased susceptibility to complications associated with chronic illnesses is common in this age group. In older individuals, poor financial condition, having multiple comorbidities, reporting chronic pain, gender (women being at higher risk), and having mental disorders have been implicated in malnutrition [8].

Poor nutritional status is associated with an increased risk of cardiac and respiratory problems, infections, deep vein thrombosis and pressure ulcers, perioperative mortality, and multi-organ failure [9]. It has been implicated in the development and progression of chronic diseases commonly affecting older people, including osteoporosis, diabetes mellitus, and cancer [9,10].

Thus, malnutrition is considered the greatest threat to the health, well-being, and autonomy of older individuals [11].

### Risk factors for malnutrition

Various studies from different parts of the world have reported that in older adults, women generally face a higher risk and prevalence of malnutrition due to factors like socioeconomic disadvantage, greater frailty, higher comorbidity burden, and poor functional status [7,12,13]. Women were socioeconomically disadvantaged, and they reported lower self-reported health status. As compared to males, female older people had high comorbidity, high drug intake, and disability.

Edentulous old individual having problems in mastication was associated with poor nutritional status in a study [14]. Chronic pain is also a significant factor for malnutrition. Pain, fatigue, illnesses, and gastrointestinal problems may also decrease appetite and cause poor nutrition [8].

The major concern for old individuals is increased longevity with the rise of comorbidity, decline in physical ability, and loss of cognitive function [15]. Aging is a process in which an individual’s physical and mental abilities are gradually lost, and vulnerability to diseases increases in old age. Dietary habits and low-nutritious diets are also implicated for comorbidity.

Living alone is a risk factor for malnutrition, as demonstrated in a previous study [16]. In old age, more older people become financially dependent on their children. The financial burden is also increased by comorbidity, frequent hospitalization, and regular drug use.

Nutritional status of older people is influenced by a complex interaction of dietary, socioeconomic, physical, and psychological factors [17]. Malnutrition will limit daily activities and worsen comorbidities [15]. The health of older people is largely affected by lifestyle, occupation, nutrition, and environmental factors as well [18]. Increased comorbidities are independently associated with poor nutritional status. Malnutrition in old age often goes undetected, and timely nutritional screening is crucial for early intervention [19,20].

### Malnutrition in developed and developing countries

Information about the nutritional status and health status of the older individuals will be of public health importance because of the growing older population, and different aspects of older people’s health, socioeconomic status, and social indicators need to be explored. A status report published by the Nepal Geriatric Centre in 2010 highlighted the research gap regarding health status and nutrition [6] and recommended research in the area in Nepal.

Reviewing 25 previous studies, mostly from developed countries (n = 14,149) on the nutritional status of the older population, Guigoz et al [21] reported a mean prevalence of malnutrition among community-dwelling older individuals that ranged between 0 and 8%, and at risk of malnutrition of 45% from developed countries. A higher prevalence of malnutrition was found in hospitalized older patients (20%), and older people residing in institutional homes (37%).

Domit et al [22], in a study on nutritional and health status among nursing home residents in Lebanon, reported a gender difference in health, socioeconomic factors, and nutritional status. The malnutrition rate was 3.2%, and the at-risk-of-malnutrition rate was 27.6%.

In developing countries, this prevalence was higher in community-dwelling older adults. Previous studies conducted in India [23] and Bangladesh [7] have revealed such proportions of malnutrition in older adults. Similarly, in Nepal, a study found that 31% of older people in Pharping VDC, near Kathmandu, were malnourished, and 51% were at risk of malnutrition [24].

### Rationale of the study

Although malnutrition is more prevalent in low-income countries where health conditions are worse among older adults due to low socioeconomic status and health-related factors [12], unfortunately, little is known about the characteristics and the needs of these older people. The situation of old aged individuals’ physical health, mental health, and social health in Nepal is largely unexplored. Studies to explore the various aspects of health characteristics, social indicators, and nutritional status of the older population in Nepal are very limited.

Between 2001 and 2011, the Lalitpur district of Nepal experienced an average annual growth rate of 3.32% with an increased older population [25]. The urban areas of the Lalitpur district were chosen for the study because, the district had a unique blend of increased population growth, rapid urbanization, and rich cultural heritage. It was an accessible setting for studying urban challenges and public health issues posed by modernization and lifestyle changes among the older adults, and no prior study was conducted on older people’s nutritional status using MNA in the area.

There were gaps in knowledge regarding the health and nutritional status of older adults in Nepal, as identified in the Status Report on Elderly People of Nepal [6]. The Nepal Demographic and Health Survey (NDHS) [26] assessed the nutritional status of adults up to the age of 49 years using BMI and did not assess the nutritional status of older people. The purpose of the study was to identify the status of nutrition and common health characteristics, such as comorbidity, hospitalization, and insomnia among community-dwelling older adults in Nepal.

### Research questions

What proportion of the older population was malnourished, and what variables were associated with malnutrition?

What percentage of older people were having common health issues such as comorbidity, self- rated health status, chronic pain, oral health problems, insomnia, and recent hospitalization?

### Objective of the study

An increased understanding of the factors that contribute to poor nutrition in older adults should enable the development of appropriate preventive and treatment strategies and improve the health of older people. The objective of the study was to assess the nutritional status, socioeconomic factors, and health characteristics among community-dwelling older people in urban areas of the Lalitpur district, Nepal.

## Methods

### Study design

The design of the study was a cross-sectional, population-based study among older people aged 60 years or older in urban areas of Lalitpur district. A sample of 360 old individuals from areas located on the outskirts of Lalitpur Metropolitan City was taken for the study.

### Sample size

The sampling technique used was multi-stage cluster sampling with probability proportional to size. The sample size was established according to the prevalence of malnutrition among home-living older people. A review of the literature on MNA for the prevalence of malnutrition among the community-dwelling old aged population (21 studies) revealed 2% malnutrition and 24% at risk of malnutrition in developed countries [22]. Studies among hospital outpatients and home care old individuals showed a higher prevalence of malnutrition (9%) and at risk of malnutrition (45%) [21]. A study found 26% malnutrition among rural older people in Bangladesh using MNA to assess nutritional status [7]. In a VDC near Kathmandu Valley in Nepal, using MNA to assess malnutrition found that 31% of the old-aged population were malnourished and 51% were at risk of malnutrition [24].

The sample size was calculated using Epi Info version 7 (Statcalc) and the formula for a population survey. Taking an expected frequency of 31% with a confidence limit of 7% and a design effect of 2, from 30 clusters, 360 participants were necessary to establish a 95% confidence interval.

### Study setting

The study site was the surrounding areas of Lalitpur Metropolitan City of Lalitpur District, Nepal. This study used the old administrative division of Lalitpur District prior to the federal restructuring of Nepal for the sampling purpose and mentioned former VDCs that were later merged as municipalities in 2017 [25].

**Fig 1:**
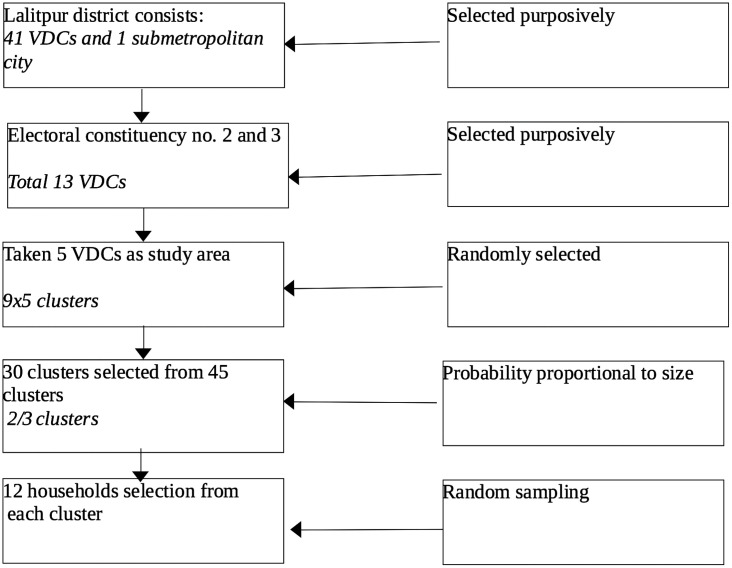
Sample selection flow chart.

The district is divided into three electoral constituencies. Populated Electoral Constituencies No. 2 and 3, consisting of 13 VDCs, were selected purposively. From the 13 VDCs, 5 VDCs (Lele, Siddhipur, Thaiba, Saibu, and Khokana) were selected randomly. Each VDC was divided into 9 wards, and each ward was taken as a cluster. In total, 45 clusters from 5 VDCs, 30 clusters were selected according to probability proportionate to size. From each cluster, 12 older individuals were selected randomly. Sample selection process is shown in the flow chart (Fig 1).

Only one older individual was selected from each household based on their willingness to participate and availability. In case of refusal to participate or unavailability at the time of data collection, another older individual from an adjacent household was selected as a replacement. The inclusion criteria of the study were participants aged 60 years or older who consented to participate in the study and did not have a severe mental illness. Severe mental illness was considered on the basis of a physician’s diagnosis and an evident mental disorder resulting in serious functional impairment, substantially interfering with major life activities such as self-care.

### Ethics and consent

Ethical approval was obtained from the Institutional Review Committee (IRC) at the Institute of Medicine, Maharajgunj Medical Campus, in Kathmandu, Nepal. The purpose of the study and data collection, including anthropometric measurements, were explained to the participants. Written consent was obtained from all participants. Participants were fully informed of their rights to decline or withdraw from participation in the study if desired. The information collected was kept confidential.

### Study tools

#### Study questionnaire.

A multi-component structured questionnaire including assessment tools was used to collect data in the study. The interviewers consisted of three trained personnel, having previous experience in field surveys and research. The questionnaire was translated into the Nepali language from English and then back-translated into English by two persons fluent in both languages. Pre-testing of the questionnaire was done in a sample of 30 older people to identify the feasibility of the questionnaire. According to the feedback, the questionnaire was revised for clarity with minor grammatical changes. Each interview took 35 minutes on average. In case of the inability to communicate effectively due to language or speech leading to difficulty in understanding, and for in need of clarification of the provided information, the help of a family member was taken. The survey was conducted from 20/09/2014 to 07/11/2014.

Although there is a time gap between the start of the study and the time of publication due to various circumstances, this study provides the status of malnutrition and health characteristics in the urban population of older adults around the Kathmandu Valley and serves as a baseline result for comparison with similar studies in the future. Policies and programs on the growing older population in Nepal are not fully data-driven. Moreover, this study provides a reference to researchers and program and policy makers. In addition, care has been taken in the interpretation and making comparisons in the article.

### Assessment tools

#### Sociodemographic factors.

Demographic characteristics included in the study were age, gender, ethnicity, religion, place of residence, marital status, and living condition (living alone or living with others). Socioeconomic status was assessed by a weighted wealth index method used by the Department of Health Survey [27]. The index assessed housing characteristics including floor, wall, and roof materials; ownership of agricultural lands; and household assets including television, electricity, telephone, motorcycle, fan, refrigerator, and computer in the house. Availability and use of drinking water for the household, use of fuel for cooking, and use of a toilet were also assessed by the index. All the items were dichotomized, and factor analysis was done by principal component analysis to reduce the items and extract factor 1. This procedure first standardized the indicator variables; then, the factor coefficient scores (factor loadings) were calculated. For each household, the indicator values were multiplied by the factor loadings as item weights and summed up to produce the household’s wealth index value. Then, the total wealth index score was divided into five categories: lowest, second, middle, fourth, and highest income groups [28].

Ethnicity was classified into groups: Brahmin/Chhetri, Newar, other Janajati (Magar, Tamang, Gurung), and Dalit.

Financial dependency on children was assessed by asking a question with three answers: “totally dependent,” “partially dependent,” and “independent.” Those older individuals who had to fully depend on their children financially for their health care expenses, daily living expenses, or for other needs, were considered totally financially dependent. Partially dependent individuals were those having some income source such as pensions but not sufficient financially, and independent individuals were those having their own sufficient income or businesses. Education was categorized as: 1) illiterates (unable to read and write), 2) informally literate, 3) Primary education, and 4) Secondary education and above (included those who had completed primary education, those having SLC, and a university degree). Main occupation was recorded based on current occupation as farmer, laborer, self-employed/job, and housework/no work. Type of family was categorized into 3 groups: nuclear, joint, and extended family.

### Nutritional status and anthropometric measures

To assess nutritional status, the MNA tool was used. It is an instrument that allows for an objective assessment of nutritional status in older people. This assessment tool is widely used in older people and has been translated into more than 20 languages. It is a sensitive tool cited in various publications. Since, the MNA tool has already been validated in various settings and proven to be a sensitive tool, a validation study was not done in our study. However, Ghimire et al. [13] conducted a validation study on the MNA in the Nepalese older population and found it to be a sensitive and reliable tool to assess the nutritional status in older adults in Nepal.

The MNA includes 18 questions grouped into 4 parts:

1) Anthropometric assessment: BMI calculated from weight and height, weight loss, and arm and calf circumferences; 2) General assessment: lifestyle, medication, mobility, and presence of signs of depression or dementia; 3) Short dietary assessment: number of meals, food and fluid intake, and autonomy of feeding; 4) Subjective assessment: self-perception of health and nutrition. Each answer has a score, and the maximum MNA score is 30. The MNA was translated into Nepali carefully to preserve the original meaning of the MNA questions and reviewed by the researcher and field interviewers. The MNA is a nutritional status assessment tool with a reliable scale and clearly defined thresholds. The MNA score of each participant was calculated, and scores were categorized as < 17 points for malnutrition, 17–23.5 for nutritional risk, and ≥ 24 for well-nourished.

Body weight was measured in light clothes and no shoes by electronic digital scale from Microlife company- Switzerland (model no. WS 50) to the nearest 0.1 kg, and height was measured with a measuring tape mounted on a wall to the nearest 0.5 cm. Measurement was taken in standing position without shoes, feet put together with heels, buttocks, shoulder, and back of the head touching the wall, and head facing straight forward. Body mass index (BMI) was calculated with the formula: Weight (kg)/ height (m^2^). Mid-upper arm circumference was measured with a non-elastic tape on the relaxed arm. Calf circumference was measured with a non-elastic tape on the thickest part of the undressed calf, with the individual sitting and their knee flexed at a 90-degree angle.

### Health characteristics

Health status was assessed by self-reported health (SRH). The 5-item self-rated health (SRH) scale was categorized into poor health, fair health, and good health. SRH was measured by asking the question, “In general, how do you describe your health?” Answers ranged between poor health, fair health, and good health. The measures were commonly used in the research and have been proven to be valid and reliable [29,30]. Self-reported co-morbidities were recorded based on physician-diagnosed chronic diseases (hypertension, diabetes, heart disease, stroke, cancer, arthritis, liver or gall bladder problem, respiratory problem, and no chronic diseases). Responses were again recorded in categories: No chronic illness, one chronic illness, two chronic illnesses, and three or more chronic illnesses. Daily drug intake was recorded as the number of drugs taken daily as prescribed by a physician. Chronic pain, defined as participants feeling pain for at least 3 months, was determined by asking a yes/no question. Participants were asked about insomnia and responses were categorized into two: no or occasionally, and often or always. Recent hospitalization, defined as hospitalization within 1 year, was recorded as yes or no.

Oral health status was assessed by yes/no questions in three domains: having chewing problems, having loss of dentition (no loss, partial loss, and total loss), and wearing dental prostheses. Tobacco smoking was assessed by a yes/no question asking whether the old aged participant was currently smoking or not smoking. Current smoking was defined as the participant having smoked within the past month. Physical exercise was assessed by asking if the individual had been exercising daily in any kind of exercise, ranging from mild brisk exercise to running, playing, or going to the gym for 30 minutes for over a year.

### Statistical analysis

Data editing and coding was done by the researcher every day. Data entry was done in EpiData 3.1. Data analyses were carried out using the Statistical Package for the Social Sciences (SPSS) software, version 20.0. Percentages were used to present nominal variables, while means and standard deviations were calculated for continuous variables. Chi-square tests were performed to assess associations between independent variables (socio-demographic factors, health characteristics) and nutritional status. An ANOVA test was performed to compare means.

## Results

From multistage cluster sampling, a sample of 360 participants was selected for the study. Among the selected older people, 6.11% refused to participate in the study and were replaced. The age and gender of the participants who refused and those who were replaced did not differ significantly.

### Sociodemographic characteristics

Table 1 presents the sociodemographic characteristics of the older people in the study. The study sample included 183 women (50.8%) and 177 men (49.2%). The mean age was 71.2 (SD = 8.2) years and was similar in both genders. Ethnicity-wise, nearly 68% of the participants were Newar and 26.9% were Brahmin/Chhetri.

**Table 1:** Socio-demographic characteristics of the study sample.

Variables		Frequency (n = 360)	95% CI
		n (%)	
**Age class**	60-70	200 (55.6)	49.5-60.8
	71-80	101 (28.1)	23.3-33.1
	>80	59 (16.4)	12.8-20.0
**Gender**	Male	177 (49.2)	44.4- 53.9
	Female	183 (50.8)	46.1- 55.6
**Ethnicity**	Newar	243 (67.5)	63.1-72.5
	Brhamin/Chhetri	97 (26.9)	21.7-31.4
	Janjati (other)	14 (3.9)	1.9-5.8
	Dalit	6 (1.7)	0.6-3.1
**Marital status**	Married	212 (58.9)	53.9-63.9
	Widowed	148 (41.1)	36.1-46.1
**Main occupation**	Agriculture	258 (71.7)	67.5-76.9
	Employed/business	51 (14.2)	10.6-18.1
	Housework	51 (14.2)	10.8-17.5
**Living condition**	Living alone	3 (0.8)	0.0-1.7
	Living with other	357 (99.2)	98.3-100.0
**Financially dependent on children**	No	131 (36.4)	30.8-40.5
	Partially	137 (38.1)	33.3-42.8
	Totally	92 (25.6)	21.4-30.6

Among the participants, 67.8% were illiterate, 14.4% did not have formal education but were literate, 6.9% had completed primary level education, and 10.8% had secondary level or higher education. About 85.8% of older people were living in a joint family, and 0.8% of the participants were living alone. Regarding main occupation, 71.7% were farmers by occupation, 14.2% were employed or having their own business, and another 14.2% had no job, doing housework.

### Nutritional status and health related characteristics

Table 2 shows the nutritional status and health-related characteristics of the study population. According to MNA, 13.1% of the study population were malnourished (MNA score < 17). The mean MNA score was 22.15 (SD = 4.23). The mean BMI was 23.09 (SD = 4.07). The underweight older population had a BMI < 18.5.

**Table 2:** Nutritional status and health related characteristics of the study sample.

Variables		Total (n = 360)	95% CI
		n (%)	
**MNA** **Mean(SD)**	Malnutrition (<17)	**22.15 (4.23)**	9.7-16.4
	At risk of malnutrition (17–23.5)	47 (13.1)	40.3-50.6
	Normal (>24)	162 (45.0)	36.7-47.2
		151 (41.9)	
**BMI** **BMI mean** (SD)	Underweight (<18.5)	**23.09(4.07)**	8.3-15.0
	Normal weight (18.5–24.99)	42 (11.7)	54.4-64.7
	Over weight (25–29.99)	216 (60.0)	19.2-28.1
	Obesity (>30)	84 (23.3)	3.1-7.2
		18 (5.0)	
**Self rated health(SRH)**	Good	121 (33.6)	28.3-38.3
	Average	208 (57.8)	52.5-63.3
	Poor	31 (8.6)	6.1-11.7
**Number of chronic diseases**	No disease	136 (37.8)	32.92- 42.89
	≤ 2	212 (58.9)	53.6- 64.0
	> 2	12 (3.3)	1.7-5.3
**Daily drug intake**	No drug	170 (47.3)	41.9- 52.5
	≤ 3	146 (40.5)	35.4-45.8
	> 3	44 (12.2)	9.2-15.8
**Chronic pain**	Yes	67 (18.6)	14.7-22.8
	No	293 (81.4)	77.2-85.3
**Hospitalization during last year**	Yes	48 (13.3)	9.7-16.7
	No	312 (86.7)	83.3-90.3
**Insomnia**	Always/Often	103 (28.6)	23.9-33.6
	Occasionally/No	257 (71.4)	66.4-76.1

The 5-item self-rated health (SRH) scale was categorized into poor health (bad or very bad health), fair health (average health), and good health (very good or good health). One-third of the participants reported their health status as good health. More than half of the study population reported their health as an average condition, and about 8.6% reported that their health condition was poor. About 37.8% reported having no chronic diseases, and 3.3% had more than two chronic diseases. Nearly 59% reported one or two chronic diseases. Hypertension was the most prevalent disease (26.5%), followed by Diabetes Mellitus (12.5%), Chronic Obstructive Pulmonary Disease (COPD)/Asthma (12.0%), arthritis (10.5%), Cardiovascular Diseases (2.3%), stroke (1.0%), and cancer (0.5%).

Nearly 34% were currently smoking tobacco. Regarding physical exercise, 25% of the participants were regularly exercising, and there was no significant difference between men and women on smoking and physical exercise.

About 12.2% of individuals reported taking more than 3 prescribed medicines daily. About 18.6% of individuals reported having chronic pain (feeling pain for at least 3 months). About 28.6% reported having insomnia always or often. About 13.1% of the individuals were hospitalized in 1 year. Regarding oral health status, 46.4% reported having a problem chewing, and 83.6% were partially or totally edentulous.

### Nutritional status, socioeconomic status, and health characteristics

This section shows the association between the independent variables and the dependent variable, i.e., nutritional status in bivariate analysis. Table 3 shows the results of sociodemographic factors and their association with nutritional status. Age was significantly associated with poor nutritional status (p = 0.04). Individuals with farming as their occupation were more often malnourished (p = 0.02). No significant association was demonstrated between independent variables such as gender, education, ethnicity, living conditions, marital status, and comorbidity. Wealth quintile and financial dependency were not significantly associated with malnutrition in the study.

**Table 3:** Nutritional status and sociodemographic factors.

Variables	Total (n = 360)	Malnourished 47, 13.1%	Risk of malnutrition 162,45.0%	Well nourished 151,41.9%	Test statistics	p-value
**MNA mean (SD)**	22.15(4.23)	14.56(2.36)	20.80(1.92)	25.97 (1.62)	747.41^a^	<0.001
**BMI mean (SD)**	23.09(4.07)	18.92(2.52)	22.40(3.98)	25.13 (3.24)	166.92^a^	<0.001
**Age mean** (SD)	71.21(8.25)	73.00(9.21)	71.60(8.59)	70.21(7.45)	2.43^a^	0.088
		**n (%)**	**n (%)**	**n (%)**		
**Age class, n(%)**					9.94	0.041
60-70	200	19(9.5)	88(44.0)	93(46.5)		
71-80	101	16(15.8)	43(42.6)	42(41.6)		
>80	59	12(20.3)	31(52.5)	16(27.1)		
**Gender, n(%)**					2.75	0.252
Male	177	26(14.7)	72(40.7)	79(44.6)		
Female	183	21(11.5)	90(49.2)	72(39.3)		
**Ethnicity n(%)**					9.02	0.172
Brhamin/Chetri	97	15(15.5)	47(48.5)	35(36.1)		
Newar	243	29(11.9)	103(42.4)	111(45.7)		
Janjati (other)	14	1(7.1)	10(71.4)	3(21.4)		
Dalit	6	2(33.3)	2(33.3)	2(33.3)		
**Living status n(%)**					0.76	1.000
Living alone	3	0(0)	2(66.7)	1(33.3)		
Living with other	357	47(13.2)	160(44.8)	150(42.0)		
**Marital status n(%)**					2.36	0.307
Married	212	26(12.3)	90(42.5)	96(45.3)		
Widowed	148	21(14.2)	72(48.6)	55(37.2)		
**Education n(%)**					11.12	0.085
No education	224	31(12.7)	115(47.1)	98(40.2)		
Literate (informally)	52	7(13.5)	27(51.9)	18(34.6)		
Primary school	25	6(24.0)	8(32.0)	11(44.0)		
Secondary and above	39	3(7.7)	12(30.8)	24(61.5)		
**Wealth quintile, n(%)**					10.10	0.258
Lowest	72	11(15.3)	30(41.7)	31(43.1)		
Second	72	8(11.1)	34(47.2)	30(41.7)		
Middle	72	11(15.3)	27(37.5)	34(47.2)		
Fourth	72	5(6.9)	32(44.4)	35(48.6)		
Highest	72	12(16.7)	39(54.2)	21(29.2)		
**Financial dependency, n(%)**					8.07	0.089
No	92	10(10.9)	33(35.9)	49(53.3)		
Partially	137	16(11.7)	70(51.1)	51(37.2)		
Totally	131	21(16.0)	59(45.0)	51(38.9)		
**Main occupation, n(%)**					10.95	0.027
Agriculture	258	31(12.0)	120(46.5)	107(41.5)		
Employed/business	51	3(5.9)	26(51.0)	22(43.1)		
Housework	51	13(25.5)	16(31.4)	22(43.1)		

Abbreviations: SD, standard deviation; BMI, body mass index; MNA, mini nutritional assessment.

^a^ANOVA test for mean differences between nutritional status groups. All others are Chi-square tests.

### Health related characteristics and Nutritional status

Table 4 shows the results of analysis for health-related characteristics and nutritional status in bivariate analysis. Regarding health-related characteristics, individuals having insomnia and hospitalization during the last year were more often experiencing malnutrition. No significant association was demonstrated between independent variables such as having more chronic illnesses, multiple drug intake, and having chronic pain, and nutritional status.

**Table 4:** Nutritional status and health related characteristics.

Variables	Total (n = 360)	Malnourished 47, 13.1%	Risk of malnutrition 162, 45.0%	Well nourished 151, 41.9%	Test statistic^c^	p-value
**Number of diseases, n(%)**					4.62	0.328
No diseases	136	18(13.2)	59(43.4)	59(43.4)		
≤ 2	212	28(13.2)	94 (44.3)	90(42.5)		
> 2	12	1(8.3)	9(75.0)	2(16.7)		
**Daily drug intake, n(%)**					4.61	0.330
No drug	170	22(12.9)	70(41.2)	78(45.9)		
≤ 3	146	17(11.6)	69(47.3)	60(41.1)		
>3	44	8(18.2)	23(52.3)	13(29.5)		
**Chronic pain, n(%)**					5.00	0.082
Yes	67	10(14.9)	37(55.2)	20(29.9)		
No	293	37(12.6)	125(42.7)	131(44.7)		
**Insomnia, n(%)**					39.08	≤ 0.001
Always/Often	103	29(28.2)	51(49.5)	23(22.3)		
Occasionally/No	257	18(7.0)	111(43.2)	128(49.8)		
**Hospitalized in one year, n(%)**					10.52	0.005
Yes	48	13(27.1)	21(43.8)	14(29.2)		
No	312	34(10.9)	141(45.2)	137(43.9)		
**Oral health, n(%)**					3.03	0.220
**Chewing problem**					3.35	0.187
Yes	167	26(15.6)	78(46.7)	63(37.7)		
No	193	21(10.9)	84(43.5)	88(45.6)		
**Edentulous**						
Yes	301	40(13.3)	141(46.8)	120(9.9)		
No	59	7(11.9)	21(35.6)	31(52.5)		

^c^All statistics are Chi-square tests.

## Discussion

This study assessed the nutritional status of community-dwelling older individuals and socio-demographic factors, health status, and social indicators on nutrition. The study revealed a high prevalence of poor nutritional status and health status among older people. The main findings of the present study were 13.1% malnutrition and 45.0% at risk of malnutrition among the older adults, as measured by MNA. Approximately 62% of participants reported having chronic diseases. About 16% of people were having chewing problems due to poor dental health. Age group, occupation, hospitalized during one year, and having insomnia were all associated with malnutrition.

### Nutritional status

The present study revealed a prevalence of 13.1% malnutrition and 45.0% at risk of malnutrition among the older adults. BMI data showed 11.7% of the elderly population was underweight, similar to the MNA malnutrition assessment. A study in a village of Nepal assessed malnutrition using MNA and showed that 24% of older people were malnourished and 65% were at risk of malnutrition [13]. A cross-sectional study in a rural VDC of Nepal revealed that 11.6% of the population was malnourished and 49.7% were at risk of malnutrition [19]. These findings are similar to our study.

A higher malnutrition rate was found in the urban area of the Terai region of Nepal. Sharma et al. [31] found a prevalence of 19.8% malnutrition and 45.7% at risk of malnutrition among older adults using the MNA tool in urban areas of the Terai region of Nepal. In the NDHS survey in 2022, BMI was used to assess the nutritional status in the population, and a higher prevalence of undernutrition was observed in this region of Nepal [32].

Minimum dietary diversity is an indicator of diet diversity and healthy eating. Having minimally adequate dietary diversity is an indicator for micronutrient adequacy [33]. More dietary diversity was observed in urban areas, Bagmati province of Nepal, and lower diversity was seen in rural areas with the lowest in Madhesh Province (Terai region). Additionally, high proportions of anaemia were observed among women in the Terai region as well [32]. This might be the reason for the higher prevalence of malnutrition in rural areas and the Terai region of Nepal. A study carried out in villages nearby Kathmandu valley, Pharping VDC, among the older population using the MNA revealed that 31% of older people were malnourished and 51% were at risk of malnutrition [24].

The lower malnutrition rate seen in our study may be due to the urban population of older people in the study. Higher minimum dietary diversity has been observed in urban areas of Nepal [32]. Another reason may be the variation in dietary practices in different cultures. In our study, the majority of the study population was Newar ethnic groups, which have a culture of eating a variety of foods, including beans and protein-rich foods. Another aspect of the Newar community is their abundant community celebrations and the variety of food served in the gatherings. This might have lowered the rate of malnutrition in our study.

In Nepal, studies have revealed a malnutrition rate of older adults ranging from 10 to 31% [13,19,20,24]. While reviewing the situation of older people’s malnutrition status in neighboring countries, a higher prevalence of malnutrition was reported in a study of rural Bangladesh among older people [7]. In this sample, MNA was used to assess the nutritional status, and the prevalence of malnutrition was 26%, and 62% were at risk of malnutrition. A lower rate of malnutrition among older adults was observed in a rural population of South India [14]. This study reported a prevalence of malnutrition of 14% and at risk of malnutrition of 49%, using MNA to assess the nutritional status among older persons aged 60 years and above. Moreover, a lower prevalence of malnutrition in community-dwelling older adults was observed in developed countries, as nutritional status was assessed by the MNA tool. In a review of 24 studies with more than 30,000 older adults worldwide, Guigoz et al. [21] reported that the mean prevalence of malnutrition among community-dwelling elderly was 1%. A higher prevalence was found in outpatients (4%), hospitalized elderly (20%), and among elderly in institutional homes (37%). Similarly, Kaiser et al. [34] found a prevalence of malnutrition of 5.8% and that 31.9% were at risk of malnutrition in a pooled result of data from five countries, including 964 community-dwelling elderly individuals. In this analysis, the authors found 22.8% malnutrition and 46.2% at risk of malnutrition in 24 combined datasets (hospital, nursing home, community, and rehabilitation settings), including information on more than 6000 elderly study participants from different settings, including home-living older adults. In a cross-sectional study among rural community-dwelling older people in Lebanon, Beulos et al. [12] found a prevalence of 8% malnutrition and 29.1% at risk of malnutrition.

In developing countries, malnutrition is a prevalent condition among older people. In Nepal, a cross-sectional study [35] revealed 13% malnutrition, measured by BMI (BMI < 18.5), among home-living older individuals. However, the use of BMI as the only tool to detect malnutrition becomes unreliable [36]. The MNA scores have been found to be significantly correlated to nutritional intake (for energy, carbohydrates, fiber, calcium, vitamin D, iron, vitamin B6, and vitamin C) anthropometric and biological nutritional parameters such as albumin, transthyretin, transferrin, cholesterol, retinol, alpha-tocopherol, and 25-OH cholecalciferol zinc [37].

The high prevalence of malnutrition or risk of malnutrition indicates the multidimensionality of malnutrition in old age [2]. Research has shown that multiple complex factors are in interplay for the development of malnutrition [38].

### Socioeconomic factors

Old age itself is a risk factor for malnutrition, as has been reported in the literature. Anorexia of aging is a cause of malnutrition in older persons, including other factors such as multiple pathologies, functional impairment, and decreased food intake [39,40]. Anorexia of aging causes decreased appetite and decreased feeding, leading to malnutrition [9]. The present study found a significant association between increasing age and malnutrition. Our study showed almost doubled malnutrition (20%) in those aged 80 years and above compared to 10% among those aged 60–70 years. This is consistent with other studies.

Low educational status is associated with poor nutritional statusin previous studies. In this study, the illiteracy rate among the older population was 62.2%, which was higher than the illiteracy rate of the general population observed in the National Census 2011 data of Lalitpur District [41]. Better education has been positively correlated with good nutritional status in studies [42,43].

Women had more malnutrition than men in previous studies. Low education, frequent widowhood, and increased comorbidity among women may explain this finding [44]. However, the current study did not find the association of malnutrition and gender. Women empowerment and various nutritional practices may have influenced the nutritional status in the district among older adults. Traditional knowledge and practice of ethnic foods and their consumption may have contributed to this especially in Newar ethnic groups [45].

Being financially dependent on children is a risk factor for malnutrition, as demonstrated in previous studies. Higher income was positively correlated with nutritional status. Boulos et al. [8] states “Economic factors are drivers of nutritional status.” Another unexpected finding in the current study is that financial dependency did not reveal any significant association with malnutrition. Unemployment, taking more prescribed medicines, and having more than one comorbidity were significantly associated with malnutrition in a study in Nepal [20]. Unemployment and having less financial status, lead to less access to adequate and nutritious foods. In our study, more than 99% of older adults were living with family. A possible reason may be that more than 60% of older people live with children in Nepal, and Nepalese culture is supportive of older individuals [46]. In our study, about 12.2% of individuals reported taking more than 3 prescribed medicines daily. It is plausible to think that more medicines are associated with loss of taste and appetite in older people, and comorbidity is associated with malnutrition in older adults. However, in our study in bivariate analysis, we did not observe significant associations between these variables. Since oral disorders have a significant effect on the well-being and life satisfaction of older individuals, as seen in previous studies [12,47], we did not find any association with malnutrition in this study. Approximately 15.6% of older people reported having chewing problems. The main oral problems of this population were missing teeth, and limitations in chewing ability. This may be due to better oral health conditions and availability of dental services in the community. Moreover, the oral health conditions and malnutrition in the older population should be further evaluated in the Nepalese population to assess the association between malnutrition and oral health.

### Health status

Nearly 63% of the study population had at least one chronic disease. Hypertension was the most prevalent disease (26.5%), followed by Diabetes Mellitus (12.5%), COPD/Asthma (12.0%), and arthritis. One study in Nepal found 59.3% of participants reported chronic pain, 35% reported respiratory problems, and 22% had hypertension [48]. Higher prevalence of non-communicable diseases (NCDs) in this study findings are similar to the data from the Global Burden of Disease (GBD), 2019, [49] which revealed a high prevalence of NCDs and increased comorbidity, causing an increase in death and disability rates in the population in Nepal. The estimates on morbidity and mortality in Nepali older adults revealed that the major causes of morbidity and disability were digestive (55%), musculoskeletal disorders (50%), cardiovascular diseases (35%), chronic kidney diseases (34%), chronic obstructive pulmonary disease (26%), and diabetes mellitus (22%) [50]. This shows the current trends of increased NCDs in the Nepalese population. Furthermore, even less is known about age-associated NCDs such as Alzheimer’s disease and related dementias [50,51].

A cross-sectional study in Bangladesh found that all of the participants were suffering from at least one medical condition [42]. In a study, most of the older people had some form of disease diagnosed by a doctor or health worker [52]. Various studies have reported that increased comorbidity is associated with malnutrition [7,8,36]. Comorbidity was not significantly associated with malnutrition in our study. However, about 13.1% of the individuals were hospitalized within one year, and recent hospitalization had increased malnutrition in the older people, which is consistent with other studies [12,17].

One-third of the participants reported their health status as good according to SRH.

Studies on SRH have shown the meaning of health ratings and that they have implications not just for survival but for the loss or maintenance of functional ability in the daily life of an individual. [30].

Chronic pain is also implicated in malnutrition, since 18% of the population had chronic pain in our study. Pain causes loss of appetite and loss of pleasure, leading to poor nutrition [8]. Interestingly, chronic pain was also not significantly associated with malnutrition in this study, although several studies have reported an association of poor nutritional status with chronic pain, chronic diseases, and an increased number of daily drug intakes [20,42].

About 28.6% reported having insomnia always or often. Insomnia was significantly associated with malnutrition in this study. Approximately 28% of older individuals with insomnia were malnourished. Ferede et al. [53] found a similar association of insomnia and malnutrition among older adults in their study, and our study results also support this finding.

### Strengths and limitations of the study

This study has some major strengths. We performed a community-based study, which provides unique information on the health and nutrition of the older population in Nepal. This study helps to fill the gaps in our knowledge on the health and wellbeing of older adults. To our knowledge, this is the first study that assessed nutritional status among older adults using MNA that has not been previously explored in this population. This study also offers insight that is applicable to other developing nations facing similar geographical, socioeconomic or health challenges among older adults.

Some limitations have to be considered in this study. Due to its cross-sectional nature, causality could not be determined. Various measures were self-reported, so recall bias may have occurred in the study. Although the age and sex of the replaced participants did not differ significantly in the study, selection bias, specifically attrition bias, may have incurred due to replaced samples. The study population is in urban areas on the outskirts of Lalitpur metropolitan city of Nepal and may not represent the population of the rural areas of Nepal.

## Conclusions

This is a study to describe the prevalence of nutritional status, health-related characteristics, and social indicators in an urban community-dwelling older population in Nepal. The study provided unique information regarding the nutritional status, health status, and social indicators of community-dwelling older people in urban areas of Nepal.

Poor nutritional status was a common condition among older people, and this study found a malnutrition prevalence of 13% among older people, and 45% were at risk of malnutrition. Along with malnutrition, an increased burden of comorbidity was observed, especially NCDs in older individuals. Insomnia, experience of chronic pain, and oral health problems were also prevalent in the study population. Since old-aged malnutrition is often undetected and underestimated, older adults’ nutritional screening should be incorporated in the national health survey. In addition, geriatrics health assessments should be regularly performed in health care facilities and communities. This study indicates an urgent need to take care of the health of the old-aged people for successful aging with timely nutritional and health interventions.

### Recommendations

Further research on a national representative sample is required to identify the regional, cultural, and gender differences in malnutrition prevalence among older individuals in Nepal. Involvement of multi-sector providers should be promoted to enhance nutritional status in older people and healthcare providers should reinforce the knowledge of older adults regarding nutrition and health. Longitudinal research is recommended to understand the direction of the causality of the findings. BMI was used in the Nepal Demographic Health Survey (NDHS) to assess the nutritional status of young adults up to 49 years of age. We recommend that the malnutrition assessment of older people, using MNA, should be included in the survey.
